# Barriers to successful treatment of alcohol addiction as perceived by healthcare professionals in Thailand – a Delphi study about obstacles and improvement suggestions

**DOI:** 10.3402/gha.v9.31738

**Published:** 2016-08-03

**Authors:** Kulnaree Hanpatchaiyakul, Henrik Eriksson, Jureerat Kijsomporn, Gunnel Östlund

**Affiliations:** 1 School of Health, Care and Social Welfare, Mälardalen University, Eskilstuna, Sweden; 2 Basic Concept of Nursing Practice Department, Boromarajonani College of Nursing Changwat Nonthaburi, Nonthaburi, Thailand; 3 Department of Nursing and Care, The Swedish Red Cross University College, Stockholm, Sweden; 4 Praboromarajchanok Institute for Health Workforce Development, Ministry of Public Health, Nonthaburi, Thailand

**Keywords:** alcohol, Delphi, policy, Thailand

## Abstract

**Background:**

Many Thai people experiencing alcohol addiction do not seek help, and those who do often have inadequate access to treatment. There are few research studies focusing on alcohol addiction treatment in Thailand.

**Objective:**

The purpose of the current study was to identify barriers to the treatment of alcohol addiction and to collect experts’ suggestions for improving treatment in Thailand. The Delphi technique was used to achieve consensual agreement among an expert panel within the field of alcohol addiction and treatment.

**Design:**

Three rounds of a Delphi survey were completed by a panel of experts in alcohol addiction, including physicians, nurses, social workers, psychologists, healthcare officers, and an Alcoholics Anonymous member. The open-ended answers provided by 34 experts in the first round resulted in 60 statements, which were later grouped into three themes. After three rounds of questionnaires, 51 statements were accepted as consensus.

**Results:**

Thirty-two experts participated in all three Delphi rounds. Over 80% of participants were particularly concerned about five obstacles to alcohol addiction treatment. The majority of suggestions from the expert panel were related to patients’ right to treatment and the national policy for reducing the negative effects of alcohol. According to the results of the present study, the experts suggested that the treatment of alcohol addiction should be continuous from primary care to tertiary care, and convenient pathways should be established in healthcare services. The experts would also like to increase the number of healthcare providers and improve their knowledge and skills in working with people experiencing alcohol addiction.

**Conclusions:**

Equal rights to health and treatment for people experiencing alcohol addiction in Thailand require policy improvements, as well as acceptance and awareness of alcohol addiction from both the public and policymakers.

## Introduction

Alcohol is a psychoactive substance with dependence-inducing properties. The degree of excessive alcohol consumption and associated problems varies widely around the world, although the burden of disease and death in most countries remains at around 3.3 million deaths worldwide per year and 5.1% of the global burden of disease is attributable to alcohol consumption (1). Moreover, alcohol consumption contributes to substantial harm, such as road traffic accidents; it can have great impact on physical and mental health and lead to economic loss (2). The World Health Organization (2010) reported that only 45.8% of countries have included alcohol addiction treatment programmes in their annual budget; in many of these countries, that allocation is shared with mental health services. Most countries use tax revenues, user fees, and/or private insurance to pay for alcohol- and drug-related healthcare and treatment (3).

In Thailand, people who consume alcohol are four times (95% confidence interval, CI 2.89–5.27) more likely to suffer from mental illness than those who do not drink alcohol. In addition, compared to abstainers, people who experience alcohol abuse are 2.6 times (95% CI 1.92–3.39) more likely to suffer from neurological diseases, 1.9 times (95% CI 1.46–2.28) more likely to suffer from gastritis, 1.7 times (95% CI 1.11–2.62) more likely to suffer from liver cancer, and 2.96 times (95% CI 2.25–3.90) more likely to suffer from liver cirrhosis (4). The most common disadvantages of alcohol abuse are reduced educational and employment opportunities, poor finances, and feeling guilt or remorse in relation to drinking habits. The most frequent problem experienced by adolescent males when drinking alcohol was fighting, while adolescent females were likely to feel guilt or remorse due to their drinking habits (5). According to Assanangkornchai et al. (6) the consumption of alcohol among adolescents was associated with risky health behaviours such as substance and drug abuse, injury-related risks, sexual activities, and suicide attempts. Thai people experiencing alcohol problems and addiction have inadequate access to treatment (7).

### The Thai strategy for reducing alcohol consumption

The Thai government implemented legislation in 2008 in order to reduce the harmful use of alcohol and to decrease the Thai people's alcohol consumption. However, the policy was weak in relation to implementing, monitoring, and providing treatment for people experiencing alcohol addiction (7, 8). Nonetheless, a positive outcome of this policy was an increased community awareness of the alcohol consumption issues among Thai people during the Buddhist rainy season reflection period. A campaign was launched by the Center for Alcohol Studies in Thailand in 2008 that focused on increasing awareness of the possible negative effects of alcohol consumption and encouraged reducing consumption or refraining from consuming alcohol entirely (7, 9).

### The suggestions for alcohol addiction prevention

An Australian study suggested that it is necessary to develop culturally specific first-aid strategies when assisting marginalized people, such as First Nations inhabitants (10). In the USA, controlling policies such as increasing prices with alcohol taxes received the highest ratings in terms of reducing binge drinking and alcohol-impaired driving in the general population as well as among youth (11). In a previous Thai study, it was suggested that policy changes were needed to improve the acceptability and accessibility of treatment for marginalized people (12).

### Barriers to accessing alcohol addiction treatment

The majority of people experiencing addiction do not disclose their addiction to healthcare providers; instead, they seek treatment for other conditions, which may be related to alcohol consumption (13). This complicates and increases the barriers to recovery for men and women experiencing alcohol addiction. One barrier is that the individual might lack awareness of the addiction problem or find it too hard to disclose based on embarrassment or shame resulting from the social stigma surrounding drinking problems (14–16). Another barrier is related to negative attitudes toward people experiencing substance and alcohol addiction from healthcare providers and that these professions might lack adequate knowledge and skills related to treating addiction (17).

The National Drug Strategy in Australia pointed out geographical and cultural barriers for alcohol addiction treatment such as language difficulties, inaccessible communities, lack of transportation or childcare, and less flexible welfare service for marginalized groups (Aboriginal and Torres Strait Islanders) (18).

Socio-economic inequality can lead to increased vulnerability and more severe alcohol problems, exacerbated by a lack of access to healthcare and other services (1). A disadvantageous social situation might also provide less motivation to seek treatment for alcohol addiction; according to a Swedish study, having ‘more to lose’ was associated with a higher motivation for recovery (19). In addition, in Thai society it has been found that the increasing magnitude of inequality has led to a greater risk of negative consequences for people experiencing alcohol problems (12); the Thailand National survey found that low-income families had more severe economic consequences from drinking than wealthy families (7). A similar conclusion was reached in the Nordic countries; furthermore, it seems that low socio-economic status was a bigger risk factor for alcohol-attributable disease than the actual level of alcohol consumption (20).

Inequity in healthcare accessibility is the intermediated causal pathway to alcohol-attributable disease and is consistent with various social determinants, including the availability of alcohol (production, distribution, regulation, and alcohol quality), drinking environment, drinking culture, gender, and the health and welfare system (21). Few research studies have been done in relation to barriers and accessibility of alcohol addiction treatment in Thailand.

## Aims

The purpose of the current study was to identify barriers to alcohol addiction treatment and to find out how experts would improve treatment for people experiencing alcohol addiction.

## Methods

The Delphi technique was utilized for achieving consensual agreement among experts within the field of alcohol addiction and treatment. These interdisciplinary experts were selected from among healthcare providers skilled in working with people experiencing alcohol addiction.

### The Delphi method

The Delphi method is utilized for collecting and condensing the opinions of knowledge in a specific area of interest. The strengths of a Delphi study based on a panel of experts are that their evaluations are more accurate and credible for the particular field under study than general opinions (22). The Delphi method was used in this study due to lack of existing research evidence related to the treatment of alcohol addiction in the Thai context (22).

### Ethical considerations

The study was approved by the Princess Mother National Institute on Drug Abuse Treatment Ethical Committee, Thailand (009/2558), and in Sweden by the Uppsala Ethical Vetting Board (2012/493). The participants received written information on the aim and procedure of the study by post before giving informed consent. The participants had the option to withdraw their participation at any time. Confidentiality and anonymity for the participants were ensured by removing the identities of the participants from transcripts and the manuscript.

### The formation of the panel

Purposive sampling was applied to find appropriate experts with a minimum of 5 years of experience in the field of alcohol addiction and currently involved in alcohol addiction treatment. These experts included researchers, nurses, health volunteers, physicians, psychologists, and social workers. The experts were identified by the Thai Ministry of Public Health, which is responsible for providing alcohol addiction treatment. The first author received the names of healthcare providers experienced in the field of alcohol addiction who were working in community hospitals, general hospitals, and specialty hospitals, covering four geographical regions of Thailand. In total 49 experts were invited in the formation of the panel.

### Sample

The experts were recruited from the South Region (*n*=4), the North Region (*n*=6), the Northeast Region (*n*=20), and the Central Region (*n*=4) of Thailand. In Round 1, 34 experts participated (26 female, 8 male), including the following delegates: nurses (24), physicians (3), psychiatrists (2), social worker (1), psychologists (2), Alcoholics Anonymous member (1), and health officer (1). The survey in the second round was completed by 33 experts and in the third round by 32 experts (see Table 1). In the third round, the majority of the experts were female (25), aged between 41 and 50 years, had 5–15 years of work experience in the field of alcohol addiction, and had an average education level of a master's degree (see Table 1).

**Table 1:** Response rate and demographic data of the panel experts, Thailand 2015

	Number		
	
	Round 1	Round 2	Round 3
Participant response rate	34/49 (70%)	33/34 (97%)	32/33 (97%)
Gender			
Male	8 (23.5%)	8 (24.2%)	7 (21.9%)
Female	26 (76.4%)	25 (75.8%)	25 (78.1%)
Age (years)			
30–40	6 (17.6%)	5 (15.1%)	5 (15.6%)
41–50	14 (41.2%)	14 (42.4%)	14 (43.8%)
51–60	14 (41.2%)	14 (42.4%)	13 (40.6%)
Work experience (years)			
5–15	25 (73.5%)	24 (72.7%)	23 (71.9%)
16–25	8 (23.5%)	8 (24.2%)	8 (25%)
26–30	1 (2.9%)	1 (3.03%)	1 (3.1%)
Education level			
Bachelor's degree	13 (38.2%)	13 (39.4%)	12 (37.5%)
Master's degree	17 (50%)	16 (48.5%)	16 (50%)
PhD	4 (11.8%)	4 (12.1%)	4 (12.5%)
Profession			
Nurse	24 (70.6%)	24 (72.7%)	23 (71.9%)
Physician	3 (8.8%)	3 (9.1%)	3 (9.4%)
Psychiatrist	2 (5.9%)	2 (6.0%)	2 (6.3%)
Psychologist	2 (5.9%)	1 (3.0%)	1 (3.1%)
Social worker	1 (2.9%)	1 (3.0%)	1 (3.1%)
Alcoholics Anonymous membership	1 (2.9%)	1 (3.0%)	1 (3.1%)
Health officer	1 (2.9%)	1 (3.0%)	1 (3.1%)
Region			
North	6 (17.64%)	6 (18.18%)	6 (18.78%)
South	4 (11.76%)	4 (12.12%)	4 (12.52%)
Central	4 (11.76%)	4 (12.12%)	3 (9.39%)
Northeast	20 (58.8%)	19 (57.57%)	19 (59.47%)

### The procedure of the Delphi study

The letter of invitation was sent to the experts by post, explaining the aim of the study and giving information about the Delphi methodology. The letter also explained that different professionals were invited, that they could respond anonymously, and that it was expected that all participants would complete all rounds of the Delphi study (estimated to be three rounds). After the experts had accepted the invitation to participate in the panel, they were received the first survey with instructions. Three Delphi rounds were conducted from January to September 2015. The deadline for completion of each round was 3 to 4 weeks. One reminder was sent to the participants 3 weeks after the initial mailing of every round.

### The three survey rounds

For the first round, a survey including five open-ended questions was sent to the 34 experts who had accepted the invitation to participate; all but one completed and returned the survey. These open-ended questions were developed by the research team based on earlier research and practical experience in the treatment of alcohol addiction. These five questions were: 1) How would you prefer to engage people experiencing alcohol addiction such that they will enter and remain in alcohol addiction treatment? 2) How would you prefer to prevent people experiencing alcohol addiction from relapsing after treatment? 3) How do people experiencing alcohol addiction respond to alcohol addiction treatment? 4) How could health services be organized and improved for people experiencing alcohol addiction? 5) What strategy should the Thai Ministry of Public Health develop for treating people experiencing alcohol addiction?

After the first round the answers were grouped, reformulated, and synthesized. All statements with similar responses were grouped together into the following three themes: 1) obstacles to alcohol addiction treatment; 2) rights to treatment for people experiencing alcohol addiction (availability, accessibility, acceptability, and quality of treatment); and 3) policies for reducing the negative effects of alcohol consumption.

The second survey round included 60 items formulated from the categorized answers in the first round, and most of these items were rated on the following scale: 1=strongly disagree, 2=disagree, 3=agree, and 4=strongly agree. In addition, two open-ended questions were included at the end of the questionnaire: 1) What are your experiences of strengths and obstacles when using the standard methods (motivation enhancement therapy, cognitive behavioural therapy, and 12-step programmes)? 2) Have you experienced any particular obstacles in caring for the different sexes in relation to alcohol addiction treatment?

The third survey round started shortly after the analysis of the second round in order to encourage all the experts to continue their participation in the study. This survey used the same scale to rate the items and was divided into two parts: the first part contained the 13 items that had not reached consensus in the second round, and the second part had 17 statements formulated from answers to the open-ended questions in the second round.

### Analysis of results

Content analysis (22) was used to analyse the five open-ended questions in the first round. Separate transcripts were written for each question by the first author (KH), who categorized the topics and provided preliminary headings for each type of statement. Similar statements were grouped together in the categories. The 167 statements were then translated into English and discussed with the co-authors (GÖ, HE, JK). Then statements with similar responses were grouped together into three overarching themes in order to create the second-round questionnaire. The second-round questionnaire and two open-ended questions were created in English, discussed with the co-author (GÖ), and translated into Thai. The structured Thai questionnaire was validated by the Thai co-author (JK) and two healthcare providers experienced in the field of alcohol addiction before being distributed to all experts.

The suggestions from each expert were extracted and listed to determine the existing obstacles and potential improvements in alcohol addiction treatment. The answers from each of the three rounds were analysed by the first author (KH) and then the research team discussed the preliminary analysis in each round before moving to the next stage. The research team also worked on the formation of each of the questionnaires to remove repetitive questions and make them easier to read.

The research team discussed preliminary findings and referred back to the open text responses for clarification whenever necessary. This process resulted in one combined list per specialty. The analysis was then performed for each of the result lists to identify any emerging themes. The themes, after being agreed upon by the team, were used as a coding framework for each of the issues under consideration, with statements belonging to a similar theme grouped together. The final stages involved identifying experts ‘consensus among themes’.

The panel's responses to the questionnaire were analysed by using frequency and percentage. Statements from each round that were rated *agree* or *strongly agree* by less than 80% of the panel were not included in the recommendations of the study and were excluded from the subsequent survey rounds. In the current study, the statements rated *agree* and *strongly agree* by 80% or more of the experts on the panel were determined to have achieved consensus. In previous studies, high levels of consensus (e.g. more than 70%) have been used to accomplish consensus of statements in the field of alcohol addiction treatment (22, 23).

The Results section includes three examples of excerpts from the open answers – responses from the open-ended questions in Round 1. The number of the Delphi round and the open-ended questions is presented in parentheses. In the excerpts omitted words are included within square brackets.

## Results

**Fig. 1:**
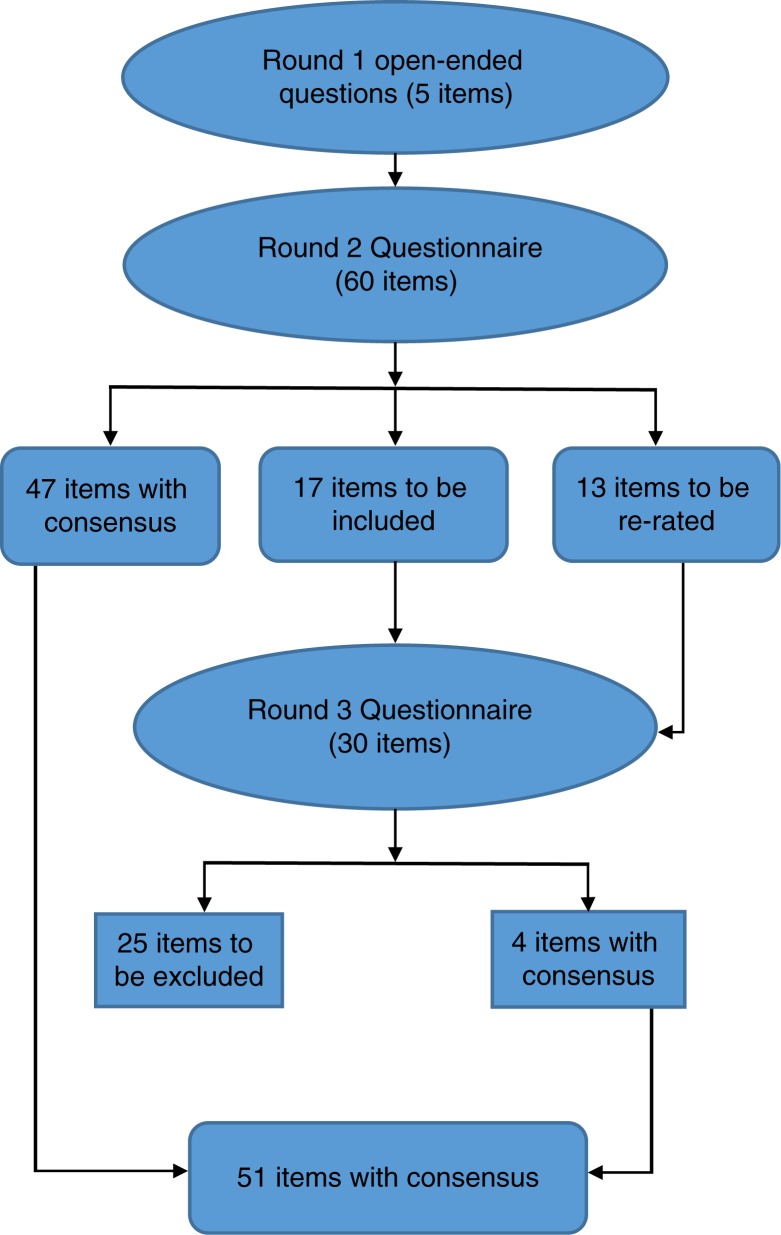
Overview of items with consensus, those excluded, and those re-rated in each survey round of the Delphi study, Thailand 2015.

In this Delphi study an expert panel from the field of alcohol addiction was used to identify barriers to accessing alcohol addiction treatment and how experts wished to improve treatment for people experiencing alcohol addiction. The results were developed through three rounds of a survey, as described in the Methods section (see Fig. 1, the flowchart of agreements). The expert panel's consensus agreements on suggested improvements and obstacles experienced were separated into three themes: general obstacles within alcohol addiction treatment and care (see Table 2), improvements in equal rights to alcohol addiction treatment (see Table 3), and general policy improvements (see Table 4).

**Table 2:** Obstacles to alcohol treatment according to the expert panel in the Delphi study, Thailand 2015

Topic	Number	Item	Agreement (%)
Obstacles to alcohol treatment	1	Patients believe that they can handle alcohol problems by themselves.	97
	2	Most patients have low motivation.	93.6
	3	There are too few healthcare providers who work with alcohol-addicted patients.	81.8
	4	Healthcare providers lack sufficient skills and education.	81.8
	5	The level of engagement by healthcare providers is too low.	81.2

**Table 3:** Equal rights to alcohol treatment with improvement suggestions from the expert panel in the Delphi study, Thailand 2015

Topic	Number	Item	Agreement (%)
Equal rights to alcohol treatment 1. Availability of alcohol treatment	1	Improve availability of screening and healthcare services throughout the entire country.	100
	2	Provide alcohol clinics in all general hospitals.	100
	3	Provide separate wards for women and men.	100
	4	Provide convenient access to alcohol treatment in hospitals, such as one-stop service.	97
	5	Provide specific wards for alcohol treatment.	97
	6	Develop continuing care in community hospitals.	97
	7	Develop continuing care in general hospitals.	97
	8	Provide training for health volunteers to identify alcohol addiction.	97
	9	Establish referral centres in general hospitals for alcohol addiction.	91
	10	Establish referral centre of alcohol addiction in community hospitals.	89
2. Accessibility of alcohol addiction treatment	11	Develop a hotline telephone service related to alcohol.	100
	12	Provide information about alcohol addiction to the public.	100
	13	Provide beds for alcohol addiction in all hospitals.	100
	14	Provide co-morbidity treatment in all treatment facilities.	94
	15	Provide aftercare centres in the community.	94
3. Acceptability	16	Promote working alliances between patients and personnel.	100
	17	Encourage patients to increase engagement in treatment.	100
	18	Encourage patients to set their own goals of sobriety.	100
	19	Promote patient-centred care.	97
	20	Develop a good attitude towards alcohol addiction among personnel.	97
	21	Promote participation by family and relatives during alcohol treatment process.	90.9
	22	Promote alcohol harm reduction goal in treatment.	84.8
	23	Consider gender differences in treating alcohol addiction.	84.8
4. Treatment quality	24	Alcohol addiction needs a specific clinical practice guideline.	100
	25	Every patient has the right to aftercare.	100
	26	The director should listen to colleagues and patients in treatment developments.	100
	27	The director of a healthcare centre should utilize evidence-based developments.	100
	28	Develop multidisciplinary teamwork to improve alcohol treatment.	100
	29	Provide proper medication for patients with multiple relapses.	97
	30	Provide Buddhist Alcoholics Anonymous in aftercare.	93.9
	31	Patients should be in the treatment process for at least 1 year.	93.9

**Table 4:** Policy improvements for reducing the negative effects of alcohol consumption suggested by the expert panel in the Delphi study, Thailand 2015

Topic	Number	Item	Consensus
Policy suggestions for reducing	1	Restrict store owners from selling alcohol to adolescents.	100
the negative effects of alcohol	2	Restrict people from drinking and driving.	100
consumption	3	Support sufficient healthcare providers to work with alcohol addiction.	100
	4	The Ministry of Public Health should promote alcohol treatment cooperation plans.	100
	5	Develop campaigns for increasing community awareness of addiction.	100
	6	Educate students and youngsters to increase self-awareness of addiction.	100
	7	Develop campaigns for increasing knowledge of addiction in work life.	100
	8	Increase the numbers of community projects for reducing alcohol consumption.	100
	9	Support adequate budget for providing more beds for alcohol addiction treatment.	94
	10	Provide compulsory treatment for alcohol addiction.	87.9
	11	Support budget for alcohol research in order to evaluate outcome of treatment.	84.5
	12	Include treatment of alcohol addiction in universal care and keep it free of charge.	84.5
Other suggestions for policy in the third survey round	1	Provide education and alcohol treatment practice for healthcare provider students.	97
	2	Provide clinical practice guidelines for each age group.	94
	3	Provide family doctor system for taking care of people experiencing alcohol addiction.	84.5

### General obstacles to alcohol addiction treatment and care

Five general obstacles were agreed upon by the expert panel (see Table 2): 1) patients lack motivation to stop drinking; 2) most patients believe that they know how to handle alcohol; 3) healthcare providers lack adequate skills and are not sufficiently educated to handle addiction; 4) understaffing in the field of alcohol addiction treatment; and 5) the level of engagement by healthcare providers is too low. For example, one expert noted the following obstacles related to alcohol addiction treatment.

The individual experiencing alcohol addiction likes to drink and lacks awareness [of addiction]. Some patients are unwilling at the first admission, and are forced by next of kin [to treatment] at the next admission; they are more able to engage in the treatment. The healthcare providers have more concern for the patient's motivation. (Round 1, open-ended question 1)

This excerpt includes mention of healthcare provider skills, forced treatment, and patient motivation. The expert panel reached consensus on two of these items: healthcare provider engagement is too low, and patients have low motivation (see Table 2). However, patients being forced to undergo treatment were close to consensus in the panel evaluation (71%). The other statements in this theme that didn't receive consensus in the third round were as follows: there are too few special wards treating alcohol addiction for women (65%), alcohol addiction treatment facilities are too far from patients’ places of residence (75%), most patients fear withdrawal symptoms (56%), and many patients’ addiction treatment has been delayed (53%).

### Equal rights to treatment for people experiencing alcohol addiction

There were 33 statements concerning equal rights to alcohol addiction treatment, which mainly comprised increased availability, accessibility, acceptability, and quality of alcohol addiction treatment (see Table 3). The majority of these statements received strong consensus agreement. The expert panel suggested that there is a need to improve the availability of alcohol addiction treatment, including the development of a well-functioning referral system with screening for alcohol problems. A suggestion from the expert panel was to train health volunteers to identify alcohol addiction problems. Moreover, alcohol addiction treatment clinics need to be present in every general hospital so people can get access to alcohol addiction treatment in every part of the country, and there should be specific beds for people experiencing alcohol addiction within co-morbidity treatment wards. To improve availability, specific wards for people experiencing alcohol addiction need to be available with separate wards for women and men. According to the expert panel, there is also a need for developing a continuing care process in relation to patients experiencing alcohol addiction. Another example from the expert's open-ended questions concerned the availability and accessibility of treatments.

The healthcare system should increase connections among primary, secondary, and tertiary care in order to provide comprehensive care for patients that covers screening, withdrawal treatment, psychosocial and co-morbidity treatment, and aftercare. It would be of value for healthcare services to share resources among the three levels and promote effective training for healthcare providers. (Round 1, open-ended question 4)

This excerpt refers to the experts’ suggestion of the need for establishing a referral centre for alcohol addiction including the alcohol addiction treatment process and training of healthcare providers (see Table 3).

The expert panel's consensus statements relating to acceptability included promoting increased patient-centred care and working alliances between patients and personnel. Moreover, the panel suggested promoting more active participation of family members in treatment. Another suggestion was to initiate alcohol harm reduction goals in treatment, as these might be more attainable for patients; even if the optimal goal is sobriety, patients must be able to set their own goals. Moreover, healthcare providers should encourage patients’ engagement in their treatment. The panel also agreed that personnel may need to adjust their attitudes towards patients experiencing alcohol addiction, including paying attention to gender differences in needs related to treatment. Another example of an expert excerpt of the open answers related to patient acceptance of the problem and of the need for help through healthcare services.

There are several factors related to individuals experiencing alcohol addiction, such as the community, family, and healthcare service. Healthcare providers and family should understand the nature of alcohol addiction and help them [patients] in an appropriate way. Patients may be ashamed and they do not want people to know about themselves; we should consider the effects of patients experiencing alcohol addiction and provide several alternatives for them. (Round 1, open-ended question 2)

In this excerpt, the statements retrieved from one expert referred to the need for healthcare staff to develop a good attitude towards people experiencing alcohol addiction and to promote the participation of families and relatives during the alcohol addiction treatment process. The expert panel reached consensus on both statements.

Several statements regarding prevention also had high degrees of consensus, including the suggestion to develop a hotline telephone service related to alcohol and to provide more information about alcohol addiction to the public. The expert panel also called for a specific clinical practice guideline for the prevention and treatment of alcohol addiction, to be prepared by the Ministry of Public Health. Moreover, the experts believed that people in treatment for alcohol addiction should remain in a continuing care process for at least 1 year. According to the panel, the treatment process should include aftercare and self-help groups such as Buddhist Alcoholics Anonymous. The panel also underlined a need for providing adequate medication for patients that had relapsed several times.

There were three statements of consensus concerning organizational developments. The panel called for more bottom-up strategies, where the director should listen to colleagues and patients in treatment. Furthermore, multidisciplinary teamwork should be encouraged to improve alcohol addiction treatment. The expert panel also asked for more developments in line with evidence-based practice. There are two statements that did not receive consensus by the panel in this theme, namely, providing follow-up treatment by telephone (68%) and the suggestion to separate alcohol addiction treatment from other substance abuse treatments (74%).

### Policy suggestions for reducing the negative effects of alcohol consumption

The expert panel agreed on the kinds of policies that are needed for reducing the negative effects of alcohol consumption in Thailand (see Table 4). Most consensus statements included restricting the sale of alcohol (e.g. to adolescents) and its consumption (e.g. no drinking and driving). The expert panel wanted the government to support an increased budget for alcohol research, treatment, and community prevention projects. Adequate funding for treatment would provide more beds for patients undergoing alcohol addiction treatment and sufficient numbers of healthcare providers. The Ministry of Public Health should promote alcohol addiction treatment cooperation plans and develop information campaigns for improving public knowledge about addiction. The other suggestions in the third round encompassed the need for providing alcohol addiction treatment education and practice for healthcare students, clinical practice guidelines, and a general practitioner system capable of providing care for people experiencing alcohol addiction.

## Discussion

This study used three rounds of Delphi study, as informed by Keeney et al. (22), to explore consensus among members of a panel with expertise related to alcohol addiction treatment in Thailand. The majority of the consensus concerned improving equal rights to alcohol addiction treatment for Thai people. The other agreements related to overcoming obstacles in treatment and policy improvements for reducing the negative effects of alcohol consumption, such as restrictions on the sale and consumption of alcohol, suggestions that reached consensus in earlier studies (11, 24, 25). Restricting store owners from selling alcohol to adolescents and inhibiting people from drunk driving were included in general policy suggestions from the World Health Organization (1) and were implemented in Thailand in 2008 (7). Although injuries and the accidents attributed to drinking and motorcycle driving have decreased in Thailand since 2004 (7), the previous alcohol policy seems to have had little effect. Thailand is ranked number two in the University of Michigan worldwide study of mortality from road accidents, with 44 road deaths per 100,000 people per year. Fatalities from road accidents made up 5.1% of Thailand's overall deaths (26); the major cause of road crashes was motorcycle driving under the influence of alcohol, with adolescents representing the biggest risk group in drunk driving (7, 27). Greater efforts are needed to counteract this problem, and this Delphi study suggests that educating the Thai public on addiction is necessary to promote awareness of alcohol's negative effects.

### Equal rights to alcohol addiction treatment

According to this Delphi study, healthcare providers’ skills and attitudes can represent barriers to providing equal access to alcohol addiction treatment for all Thai citizens. The panel agreed on three obstacles related to the lack of sufficient knowledge and skills of healthcare providers, including negative attitudes towards patients experiencing alcohol addiction. These results are consistent with a previous study showing that healthcare providers need more appropriate training to improve their knowledge of alcohol addiction and treatment (28). Other research argues that healthcare professionals’ awareness and skills surrounding alcohol addiction can be developed through education and training (29, 30). Similarly, a study from the UK suggested that training for all healthcare providers can encourage positive attitudes and that guidance for identifying and treating mental illness and substance abuse should be provided to trainees (31).

The expert panel agreed on the importance of facilitating equal rights to treatment for people experiencing alcohol addiction with the purpose of reducing alcohol consumption and alcohol-related harm. These findings were consistent with those of Panaretto et al. (32), who suggested that an individual's engagement would be promoted through screening for alcohol problems and by delivering brief intervention in primary care. The findings from the present study also reflect agreement among the experts concerning obstacles from the patients’ side, such as a lack of motivation to seek help and the belief that they already know how to handle alcohol problems. The findings of the present study are consistent with previous research showing that people experiencing alcohol addiction do not perceive themselves as being in need of help and do not seek treatment for alcohol addiction (13, 33, 34). Moreover, low levels of motivation to stop drinking distinguish those who do not seek treatment from those who do (33, 35). This is also consistent with previous research on Thai men experiencing alcohol addiction, as those who expressed low motivation during inpatient care tended to perceive medication as the most important aspect of treatment (14). However, levels of motivation to seek treatment for alcohol addiction might also be related to social situations and whether or not they have something to lose, according to severely dependent alcoholics in both voluntary and compulsory treatment (19).

Since 2002, Thailand has provided compulsory treatment for people experiencing drug addiction (though not for people experiencing alcohol addiction); the number of patients seeking drug treatment has increased since this policy was implemented (36). Major difficulties have been encountered in recruiting and retaining patients experiencing alcohol addiction in treatment, which may be related to proximity, available beds, and economic costs of travelling to treatment facilities. The experts also agreed that there should be no charge for treatment for patients with alcohol addiction throughout the entire country. However, similar difficulties recruiting patients to alcohol addiction treatment were found in reports from Australia and Finland, with increasing numbers of patients included in compulsory treatment of substance addiction (32, 37).

There are potential benefits in a healthcare system that manages to treat substance abuse and addiction. Research has argued that substance abuse treatment may help to improve access, efficiency, economy, effectiveness, and continuity of care and thereby can reduce the health burden attributable to both substance abuse and alcohol addiction (3). In Thailand, this overlapping positive effect from substance abuse treatment towards alcohol addiction treatment availability is not yet evident.

Another agreement reached by the expert panel was related to acceptability and promotion of patient-centred care, in particular the working alliance between patients and personnel. The findings are congruent with previous studies indicating that the therapeutic alliance is more likely to result in positive treatment outcomes than are specific interventions (38). Moreover, the expert panel agreed that healthcare services need to consider the effects of co-morbidity among people experiencing alcohol addiction, as well as appropriate treatment. This has been underlined previously in relation to co-morbidity treatment in different healthcare services, particularly as screening can help identify people experiencing alcohol addiction (39, 40, 41). According to nurses’ perspectives, obstacles in referring patients with co-morbidities to specialty care are related to administrative lassitude (39, 40, 41). There is currently insufficient coordination between the healthcare facilities to provide enough respect for patients’ basic human rights (3, 42).

### Methodological considerations

A strength of this study was that the Thai expert panel's response rate met the quality demands of a Delphi study, which should be at least 70% (22, 23). However, a limitation was that most of the participants came from the northeast part of Thailand, meaning that the sample did not represent all parts of Thailand to the same extent. However, the northeast parts face the heaviest burden of alcohol and drug abuse in the country.

Barriers to successful alcohol addiction treatment in the present study were mainly perceived by the majority of female nurses with a master's degree working in northeastern Thailand. However, our results are possibly transferable to other regions in Thailand, since the majority of professions working within the field of alcohol addiction are nurses (43). The Thai experts on alcohol addiction treatment in the present study were reached through the Ministry of Public Health, who picked out possible participants for the panel irrespective of educational background.

### Implications

The expert panel agreed that people experiencing alcohol addiction in Thailand encounter several barriers related to receiving adequate alcohol addiction treatment. The experts suggested that the treatment of alcohol addiction should be consistent from primary care through tertiary care, providing continuing care and information for both the family and patient. They also recommended establishing pathways within the healthcare services and increasing the number, knowledge, and skills of healthcare providers working with people experiencing alcohol addiction.

The negative consequences of excessive alcohol consumption are a global issue (1). Thailand has an increase in alcohol consumption and people do not have enough access to treatment (7, 14). Findings in the present study indicated the referral system is not well developed and phases of treatment are fragmented in between levels of care and within hospitals. The expert panel underlined the necessity of improving equal rights to alcohol addiction treatment for Thai people in all geographical regions.

## Paper context

In order to overcome the barriers to alcohol addiction treatment, the expert panel agreed to improve the equal right to alcohol addiction treatment including availability accessibility, acceptability and treatment quality of alcohol addiction treatment. In addition, the experts suggested the supporting from the political can facilitate to reduce alcohol consumption and harmful use of alcohol in the Thai context.
